# Pseudomonas aeruginosa ventriculitis following pancytopenia due to myelodysplastic syndrome with an excess of blasts type 2

**DOI:** 10.1016/j.clinsp.2023.100250

**Published:** 2023-07-18

**Authors:** Carla Alexandra Scorza, Fulvio Alexandre Scorza, Josef Finsterer

**Affiliations:** a Neurology & Neurophysiology Center, Vienna, Austria; b Disciplina de Neurociência, Universidade Federal de São Paulo, Escola Paulista de Medicina (UNIFESP/EPM), São Paulo, SP, Brazil

## Commentary

Ventriculitis as a manifestation of sepsis due to Pseudomonas aeruginosa has been repeatedly described,^1^^,^^2^ but septic ventriculitis due to Pseudomonas aeruginosa under immunosuppression due to Myelodysplastic Syndrome (MDS) with Excess of Blasts type-2 (MDS-EB2, blasts make up 10%‒19% of the cells in the bone marrow, or 5%‒19% of the cells in the blood), that progressed to Acute Myeloid Leukaemia (AML), is not reported.

The patient is an 82-year-old male with a previous history of recurrent sinusitis for 30 years, arterial hypertension, diabetes, leukoencephalopathy, hyperuricemia, gonarthrosis, prostate hyperplasia, phimosis with recurrent urinary retentions requiring recurrent urinary catheterization, recurrent urinary tract infections, and restless leg syndrome, who was diagnosed with MDS-EB2 eleven months earlier upon bone marrow biopsy. Bone marrow biopsy showed hypercellularity, depressed erythro- and granulozytopoiesis, proliferating dysplastic megakaryocytes, > 5% HLA-DR positive blasts, 16% nuclear shadows on Flow Cytometry Cell Sorting (FACS), 16% CD34- cells, and the variants JAK2 c.1849G>T, SRSF2 c.284C>A, TET2 c.4113dupC, TET2 c.3899T>G, DNMT3A c.1993G>A, DNMT3A c.856-79_1554+41del on a Myeloid Solution (MYS) panel (Sophia genetics). His medication on admission included venetoclax, tamsulosin, esomeprazol, nebivolol, allopurinol, valacyclovir, dexpanthenol, and filgastrim (recombinant Granulocyte-Stimulating Factor ‒ GCSF) every second day. He wore an indwelling catheter because of recurrent urinary retentions.

**Fig. 1:**
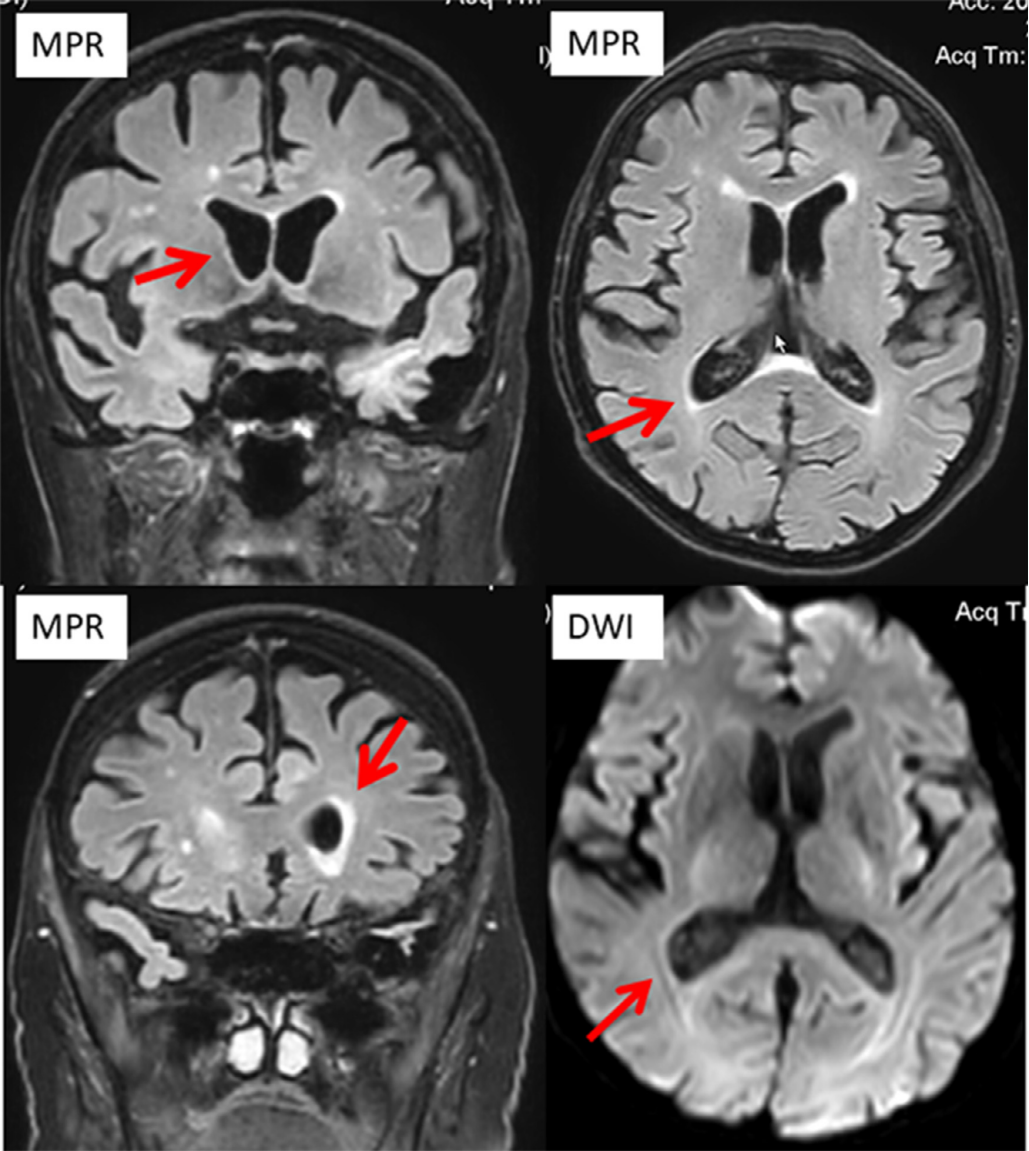
Cerebral MRI with contrast medium on hospital day 2 showing ependymal enhancement.

He was actually admitted because of recurrent dysuria starting four days prior to admission, falls, fever, and progressive confusion. ECG showed new atrial fibrillation. C-reactive protein was 331.7 mg/L (n, 0.0‒4.9 mg/L), the erythrocyte count 2.7 T/L (4.2‒55 T/L), leukocytes 0.8 G/L (n, 4.0‒10.0 G/L), and thrombocytes 89 G/L (n, 150‒400 G/L) (Table 1). There was moderate renal insufficiency and hyponatremia (Table 1). Urinary tract infection was diagnosed, and linezolid was begun on hospital day 1 (hd1). Cerebral CT on hd1 revealed only a right frontal, chronic Subdural Hematoma (SDH). A clinical neurologic exam on hd2 revealed disorientation, mild left-sided spastic hemisyndrome, recurrent cloni on the left side, and allodynia on the left thigh. Cerebral MRI with gadolinium showed ventriculitis of both lateral ventricles (Fig. 1) but had to be discontinued because of restlessness. Lumbar puncture revealed 975 cells/L (n, 0‒4 cells/L) and a protein of 689 mg/dL (n, 15‒45 mg/dL). Broad-spectrum PCR was non-informative. Cerebrospinal Fluid (CSF) culture for fungi was negative, but blood culture and CSF culture grew Pseudomonas aeruginosa. Despite adequate antibiotic (amikacin), antiviral (acylcovir), antifungal, and antiepileptic (levetiracetam) therapy the patient died from Pseudomonas aeruginosa sepsis with multiorgan failure, including the brain. An autopsy was not performed.

**Table 1:** Blood tests during hospitalisation.

Parameter	RL	hd1	hd2	hd3	hd4
CRP	0‒4.9 mg/L	296.3	321.2	nd	289.7
Leucocytes	4‒10 G/L	1.1	0.8	1.0	1.4
Erythrocytes	4.‒5‒5 T/L	3.0	2.7	3.1	3.1
Hemoglobin	14‒18 g/dL	8.8	8.0	9.0	9.1
Hematocrit	42%‒51%	23.9	21.8	24.5	25.3
Thrombocytes	150‒400 G/L	91	89	72	88
Glucose	70‒100 mg/dL	223	nd	nd	129
Creatinine	0.67‒1.17 mg/dL	1.28	1.5	1.5	1.75
GFR	90‒2001.7 m^2^BS	51	42	42	35
Sodium	136‒146 mmoL/L	122	122	128	137
Procalcitonin	0‒0.5 ng/mL	nd	10.1	nd	nd

CRP, C-Reactive Protein; GFR, Glomerular Filtration Rate; hd, Hospital Day; nd, Not Done; RL, Reference Limits.

The presented patient is interesting for ventriculitis due to Pseudomonas sepsis originating from an urinary infection. Urinary infection was presumably precipitated by MDS-EB2 with severe leukopenia despite the regular application of the GCSF filgastrim, and by urinary retention due to phimosis and a urinary catheter. MDS causes immunosuppression due to leukopenia and is known to give rise to infectious disease.^3^ Whether the application of venetoclax for MDS/AML favored the development of leukopenia or added to the immunosuppression, remains speculative, but there are concerns regarding incomplete count recovery, prolonged cytopenia, and increased infection rates, particularly when venetoclax is combined with chemotherapy.^3^

Ventriculitis refers to the inflammation of the ependymal lining of the cerebral ventricles, usually secondary to a septic infection, due to meningitis, cerebral abscess, device-related, or due to neurosurgery or trauma.^4^ Ventriculitis is not well defined and there are no universally recognized and binding diagnostic criteria.^4^ The most common presenting features are fever, headache, and fluctuating mental status.^2^ Diagnosis relies on history, clinical exams, blood tests, cerebral imaging, and CSF investigations.^4^ Treatment is based on anti-microbial agents after creating an antibiogram. Early diagnosis is crucial as it significantly improves the outcome, which depends on the causative microbial agent but is generally poor.^4^ In a recent study of 98 patients with ventriculitis, the mortality rate was 31%.^5^ Ventriculitis associated with sepsis is a known phenomenon that has been repeatedly reported.^6^ Ventriculitis in the index patient was diagnosed on hd2 but despite immediate adequate antibiotic treatment the patient did not survive.

Gram-negative bacterial infections of the Central Nervous System (CNS) are generally associated with high morbidity and mortality rates.^7^ Ventriculitis reduces CSF production.^7^ In a mouse model of ventriculitis by injecting gram-negative rods into the cisterna magna, histological examination of the choroid plexus 24 hours after the injection revealed patches of altered epithelium, with swollen and vacuolated ependymal cells associated with leukocyte infiltration.^7^ Electron microscopy demonstrated a reduced number of microvilli and flattening of the epithelial surface,^7^ suggesting that gram-negative septic ventriculitis is able to induce visible ultrastructural alterations of the choroid plexus, which are consistent with markedly reduced functioning of the epithelial choroid plexus surface.^7^ These morphological alterations could explain why the index patient had developed seizures.

In conclusion, this case shows that immunosuppression due to MDS favors recurrent urinary tract infections, which can progress to fatal septic ventriculitis. Patients with impaired consciousness during a simple urinary tract infection complicated by sepsis require immediate neurological work-up not to delay adequate treatment for CNS involvement in the sepsis.

## Authors’ contributions

JF: Design, literature search, discussion, first draft, critical comments, final approval. AS and FS: Data acquisition, literature search, discussion, critical comments, and final approval.

## Funding source

No funding was received.

## Ethical approval statement

This article is based on previously conducted studies and does not contain any new studies with human participants or animals performed by any of the authors.

## Conflicts of interest

The author declares that the research was conducted in the absence of any commercial or financial relationships that could be construed as a potential conflict of interest.
